# Molecular Detection of EMT Markers in Circulating Tumor Cells from Metastatic Non-Small Cell Lung Cancer Patients: Potential Role in Clinical Practice

**DOI:** 10.1155/2018/3506874

**Published:** 2018-02-27

**Authors:** Annalisa Milano, Francesca Mazzetta, Sabatino Valente, Danilo Ranieri, Laura Leone, Andrea Botticelli, Concetta Elisa Onesti, Salvatore Lauro, Salvatore Raffa, Maria Rosaria Torrisi, Paolo Marchetti

**Affiliations:** ^1^Department of Molecular and Clinical Medicine, School of Medicine and Psychology, Sapienza University of Rome, Rome, Italy; ^2^Oncology Unit, Sant'Andrea University Hospital, Rome, Italy; ^3^Cellular Diagnostics Unit, Sant'Andrea University Hospital, Rome, Italy

## Abstract

**Background:**

Non-small cell lung cancer (NSCLC) is the most common cause of cancer-related mortality; nevertheless, there are few data regarding detection of circulating tumor cells (CTCs) in NSCLC, compared to other kinds of cancers in which their prognostic roles have already been defined. This difference is likely due to detection methods based on the epithelial marker expression which ignore CTCs undergoing epithelial-mesenchymal transition (CTCs^EMT^).

**Methods:**

After optimization of the test with spiking experiments of A549 cells undergoing TGF-*β*1-induced EMT (A549^EMT^), the CTCs^EMT^ were enriched by immunomagnetic depletion of leukocytes and then characterized by a RT-PCR assay based on the retrieval of epithelial and EMT-related genes. Blood samples from ten metastatic NSCLC patients before starting treatment and during chemotherapy were used to test this approach by longitudinal monitoring. Ten age- and sex-matched healthy subjects were also enrolled as controls.

**Results:**

Recovery experiments of spiked A549^EMT^ cells showed that the RT-PCR assay is a reliable method for detection of CTCs^EMT^. CTCs^EMT^ were detected in three patients at baseline and in six patients after four cycles of cysplatin-based chemotherapy. Longitudinal monitoring of three patients showed that the CTCs^EMT^ detection is related to poor therapeutic response.

**Conclusions:**

The RT-PCR-based approach for the evaluation of CTCs^EMT^ phenotype could be a promising and inexpensive tool to predict the prognosis and the therapeutic response in NSCLC patients.

## 1. Introduction

The metastatic process consists in the reproduction at distance of tumor masses coming from the primary tumor cells, with a notable spatial and distrectual discontinuity from the initial localization. This process is subdivided into multiple steps that are all necessary to form a new tumor colony: loss of cell-to-cell adhesion, extracellular matrix invasion, intravasation and spreading into the bloodstream, escape from the circle, and secondary lesion establishment [1]. These structural and functional changes in the cancer tissue take place through a peculiar modulation of tumor cell phenotype known as epithelial-mesenchymal transition (EMT), a process characterized by a dramatic change of epithelial cells that lose their differentiated phenotype to acquire ex novo mesenchymal features [2]. EMT culminates with the acquisition of cell motility and invasiveness by the reorganization of the cytoskeleton dynamics such as the rearrangements of the intercellular junctions and the changes in apical-basal polarity [3].

The EMT program is sustained by hypoxia and cellular stress [4] and/or turned on by many transcription factors (TFs), including Snail, ZEB, and Twist. Working in tandem with multiple signaling pathways such as TGF-*β*, Wnt, Notch, and NF-*κ*B, their activity seems to upregulate the expression of genes related to mesenchymal phenotype (i.e., N-cadherin, fibronectin, and vimentin) and to suppress epithelial genes such as epithelial cell adhesion molecule (EpCAM), E-cadherin, *β*-*γ*-catenin, and cytokeratins (CKs) [5, 6].

Subsequently to the EMT process, cancer cells that constantly spread in the bloodstream are defined as circulating tumor cells (CTCs) [7]. It is believed that only a little part of CTCs consists of cells able to start a clonal metastatic lesion; in fact, most of them are subject to phenomena of immunomodulation and hemodynamic pressure that lead to their destruction [7–9].

Considering that CTCs are extremely rare, their identification and characterization require the application of highly sensitive and specific techniques. The pivotal technique for *in vitro* diagnostic use (CellSearch® Veridex, Menarini Silicon Biosystems) is based on the principle of immunoaffinity toward CTC surface antigens by immunomagnetic beads coated with antibodies toward EpCAM. The CTC count is a good marker for tumor growth and aggressiveness; it has been shown that a higher CTC count is associated with a decreased progression-free survival and overall survival of patients under treatment for metastatic breast [10], colorectal [11], or prostate cancer [12].

Non-small cell lung cancer (NSCLC) is the leading cause of cancer death due to distant metastases involving approximately 70% of patients who come to diagnosis [13]. The detection of CTCs in advanced NSCLC is surprisingly low with respect to other epithelial tumors [1]. In fact, the use of isolation strategies, exclusively based on epithelial marker expression, led to a CTC detection in only a third of metastatic patients [1, 14, 15] and in a very low percentage of nonmetastatic subjects [16].

CTCs are heterogeneous and are often characterized by downmodulation of epithelial markers; this feature makes the standard approaches less effective and suggests the need of an alternative detection method [17].

In this clinical setting, considering that EpCAM-based methods have low sensitivity, selection bias, and poor specificity [18], other Non-EpCAM-based capture methods have been proposed to improve CTC detection in NSCLC [19–21]; some of these are based on a negative enrichment by immunomagnetic depletion of leukocytes [22]. To minimize the leucocyte noise, density-based techniques (i.e., Ficoll-Hypaque or OncoQuick) could be used for the enrichment step before detection [23]. Then, the negative enrichment allows the recovery of the CTCs^EMT^ that can be highlighted using several techniques for the detection of EMT-related elements [24–27].

In the present study, we designed a RT-PCR approach to improve the detection of CTCs^EMT^ in metastatic NSCLC patients. To this purpose, we analyzed the peripheral blood sample for the expression of epithelial (CEA-CK19) and EMT-related genes such as vimentin and EMT transcription factors (Snail1-2, ZEB1-2, and Twist1-2). We optimized our method on A549 cells undergoing TGF-*β*1-induced EMT spiked into blood samples of healthy donors at serial dilution. This approach was subsequently tested on blood samples of ten metastatic NSCLC patients before starting treatment and during chemotherapy by a longitudinal monitoring. Ten healthy subjects, age and sex matched, were recruited in the control arm as negative controls.

Based on molecular marker expression, we identified patients with CTCs with epithelial phenotype and/or biphenotypic CTCs with expression of EMT-related genes. After defining the CTC molecular profile before treatment (T0, baseline), we analyzed whether chemotherapy induces dynamic changes and if those could be related to a response to treatment. For this purpose, we monitored the patients at the time of first posttreatment CT scans (T1) and for still living patients at the time of second posttreatment CT scans (T2).

According to our thresholds and parameters, we detected CTCs^EMT^ in three patients at baseline and in six patients after 4 cycles of cysplatin-based chemotherapy. By longitudinal monitoring, we found a poor therapeutic response in patients with CTCs^EMT^ positivity.

This exploratory study, although limited to a very small number of patients, suggests that the RT-PCR-based approach for CTCs^EMT^ detection could be a promising tool useful to predict the prognosis and the therapeutic response also in NSCLC patients.

## 2. Materials and Methods

### 2.1. Optimization of Method for Epithelial-Mesenchymal Transitioned Cell (A549^EMT^) Isolation and Characterization

#### 2.1.1. Cell Cultures and Treatments to Induce *In Vitro* EMT Phenotype

The A549 (human lung adenocarcinoma) cell line [28] was cultured in Dulbecco's modified Eagle medium (DMEM) supplemented with 10% fetal bovine serum (FBS) and antibiotics. EMT was induced by 5 ng/ml of TGF-*β*1 (PeproTech Inc., Rocky Hill, NY, USA) for 48 hours [29], and the induction of A549^EMT^ phenotype was evaluated by morphological and immunofluorescence analyses. Regarding morphological analysis, the cell cultures were studied with an Axiovert 200 inverted microscope (Zeiss, Oberkochen, Germany) equipped with differential interference contrast (DIC) optics and with Axiovision Image Analysis System (Zeiss). For the immunofluorescence analysis, the A549 cells were fixed with 4% paraformaldehyde followed by treatment with 0.1 M glycine for 20 min at 25°C and with 0.1% Triton X-100 for additional 5 min at 25° for cell membrane permeabilization. Cells were then incubated with the following primary antibodies: anti-CD326/EpCAM-PE mAb (1 : 10 in PBS; Miltenyi Biotec, Bergisch Gladbach, Germany) and anti-vimentin mAb (1 : 50 in PBS; clone V9; Dako, Glostrup, Denmark) for 15 min. The unconjugated primary antibody was visualized, after appropriate washing with PBS, by using goat anti-mouse IgG–FITC (1 : 50 in PBS; Cappel Research Products, Santa Ana, CA, USA) for 30 min at 25°C. Nuclei were stained with 4,6-diamidino-2-phenylindole dihydrochloride (DAPI, 1 : 10,000; Sigma Chemicals, St. Louis, MO, USA). Fluorescence signals were analyzed by ApoTome System (Zeiss) connected with an Axiovert 200 Inverted Microscope (Zeiss), and image analysis was performed using the Axiovision software (Zeiss).

#### 2.1.2. Immuno-Magnetic A549^EMT^ Isolation

**Figure 1:**
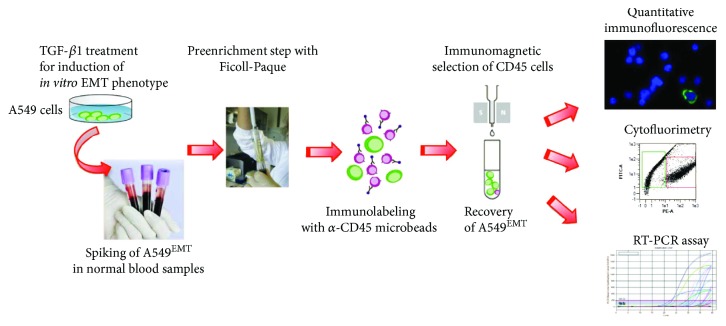
Schematic drawing of the immunomagnetic isolation and detection of A549^EMT^ cells. After negative enrichment by immunomagnetic depletion of leukocytes, putative A459^EMT^ cells spiked in the normal blood samples were analyzed with quantitative immunofluorescence microscopy, cytofluorimetry, and RT-PCR assay.

To optimize the isolation technique of CTCs^EMT^, we evaluated the recovery of A549^EMT^ cells diluted in a healthy volunteer's blood samples. We used a tenfold serial dilution (10→10^5^/7.5 mL) of cultured cells in order to determine a recovery ratio and linearity of the analysis method. In the preenrichment step, we isolated the A549^EMT^ cells using density gradient centrifugation over Ficoll-Paque™ PLUS (Amersham Biosciences/GE Healthcare, Uppsala, Sweden) recovering the PBMC layer where our cultured cells are included for physical characteristics of density. Then, the immunomagnetic negative selection helped us to clean the recovered sample from PBMC noise and to keep our epithelial and/or transition cells. To achieve this result, the PBMC was resuspended for magnetic labeling in 80 *μ*L of MACS® separation buffer (Miltenyi Biotec) and incubated for 30 min at 4°C with 20 *μ*L of CD45 MicroBeads (Miltenyi Biotec). Then, LS separation columns (Miltenyi Biotec) were equilibrated with 0.5 ml of MACS separation buffer, and the microbead-labeled cells were subjected to a magnetic field through the column. Thus, the column was removed from the magnetic separator, and the eluate was recovered. So we analyzed the eluate with quantitative immunofluorescence (qIF), flow cytometry (FC), and RT-PCR (Figure 1).

#### 2.1.3. A549^EMT^ Cell Count and Linearity Evaluation of the Methods

To evaluate the recovery ratio and the linearity of A549^EMT^ isolation method, the spiked A549^EMT^ cells were counted by qIF analysis as previously described [30, 31]. After permeabilization, the samples were incubated with anti-vimentin mAb (1 : 50 in PBS; clone V9; Dako) for 15 minutes and goat anti-mouse IgG–FITC (1 : 50 in PBS; Cappel Research Products) for 30 min and, after extensive washing, with anti-CD45-PE (1 : 10 in MACS Miltenyi Biotech separation buffer) for 15 min at 4°C. Cells were then washed and centrifuged at 1300 rpm for 6 min at 25°C, and the pellet was resuspended in 10 *μ*L of cell solution and spotted on 8-well diagnostic slides (Menzel-Glaser, Braunschweig, Germany), left to dry, and fixed with acetone for 8 min at −20°C. Nuclei were stained with DAPI (1 ng/mL, Sigma Chemicals, St. Louis, MO, USA). The A549^EMT^ were identified as vimentin^+^/CD45^−^ cells, and the cell counts were assessed independently by two pathologists.

As an additional control method of cell count, the samples of spiked A549^EMT^ were analyzed in parallel with MACSQuant Analyzer flow cytometer (Miltenyi Biotec GmbH). The excitation and emission wavelengths were 488 and 525 nm for anti-vimentin-FITC detection (B1 channel) and 488 and 585 nm for anti-CD45-PE detection (B2 channel). The fluorescence signals were analyzed by MACSQuantify software (Miltenyi Biotec GmbH), and the cells with the expression of vimentin were gated and counted.

To assess the linearity of control methods for cell count, the number of recovered cells obtained by qIF and CF was correlated by linear regression to the number of spiked A549^EMT^ cells. To verify the reliability of RT-PCR method, the expression levels of all target genes were compared by Bland & Altman plot to qIF and CF counts across all the data points. To confirm the linearity of RT-PCR assay, the expression levels of target genes were correlated by linear regression to the number of spiked A549^EMT^ cells.

#### 2.1.4. Primers

Oligonucleotide primers for target genes and for the housekeeping gene (GAPDH) were chosen with the assistance of the Oligo 5.0 computer program (National Biosciences, Plymouth, MN, USA) and purchased from Invitrogen (Carlsbad, CA, USA). The primers used are shown in Table 1. For each primer pair, we performed no-template control and no-reverse-transcriptase control (RT negative) assays, which produced negligible signals.

#### 2.1.5. RNA Extraction and cDNA Synthesis

RNA was extracted using the TRIzol method (Invitrogen) according to the manufacturer's instructions and eluted with 0.1% diethylpyrocarbonate- (DEPC-) treated water. Total RNA concentration was quantitated by spectrophotometry, and the quality was assessed by measuring the optical density ratio at 260/280 nm. RNA samples were stored at −80°C. After denaturation in DEPC-treated water at 70°C for 10 min, 1 *μ*g of total RNA was used to reverse transcription using iScript™ cDNA synthesis kit (Bio-Rad, Hercules, CA, USA) according to the manufacturer's instructions.

#### 2.1.6. PCR Amplification and Real-Time Quantitation

RT-PCR was performed using the iCycler Real-Time Detection System (iQ5 Bio-Rad) with optimized PCR conditions. The reaction was carried out in 96-well plate using iQ SYBR Green Supermix (Bio-Rad) adding forward and reverse primers for each gene and 1 *μ*l of diluted template cDNA to a final reaction volume of 15 *μ*L. All assays included a negative control, and they were replicated three times. Real-time quantitation was performed with the help of the iCycler IQ optical system software version 3.0a (Bio-Rad Laboratories), according to the manufacturer's manual. Results are reported as mean ± standard error (SE) from three different experiments in triplicate. For CEA and CK19 gene expression, the external standard was prepared as previously described [31]. To perform external standards for target genes of vimentin and EMT trascription factors (EMT-TF), tenfold serial dilutions of cDNA from A549^EMT^ cell line was used (10→10^6^ A549^EMT^ cells). For RT-PCR data analysis, the target gene expression values were corrected with housekeeping gene expression values, to target genes/GAPDH mRNA ratios. Due to background transcription in the PBMC, the cutoff levels of target genes were defined using receiver-operator characteristic (ROC) curves based on the analysis (GraphPad Prism version 5.00 for Windows, GraphPad Software, San Diego, CA, USA) of PBMC samples (true negatives) and samples of cell lines used to perform the target gene external standards (true positives).

### 2.2. Detection of CTCs^EMT^ in NSCLC Patients

#### 2.2.1. Ethic Statement, Patients, and Blood Sample Collection

Patients and healthy volunteers provided their written informed consent to participate in this study, according to a protocol study approved by the Ethical Committee of Sant'Andrea University Hospital (number 97/2012). Ten consecutive NSCLC metastatic patients treated with cisplatin-based chemotherapy were enrolled. Ten healthy volunteers age and sex matched were recruited in the control arm. The encoding of the samples was performed by the staffs of the Oncology Unit of the Sant'Andrea Hospital. In accordance with the guidelines approved by the management of Sant'Andrea Hospital for routine care purposes, 7.5 mL of peripheral blood was collected from each patient, for CTC evaluation before chemotherapy (baseline), at the time of first posttreatment CT scans (T1), and for still living patients, at the time of second posttreatment CT scans (T2). EDTA blood samples were delivered directly to the Cellular Diagnostics Unit laboratory and immediately processed to isolate the PBMC using density gradient centrifugation. CTCs were isolated by immunomagnetic negative depletion and detected with RT-PCR as described above.

#### 2.2.2. Statistical Methods

All the correlation measures in the recovery experiments and calculations of RT-PCR efficiency were evaluated by the Pearson test (*r*^2^) and by linear analysis of regression curve. To compare the analytic methods, the Bland & Altman plot was performed; the limits of agreement for interchangeability of the methods are defined as the mean difference plus and minus 1.96 times the standard deviation of the differences. Analysis of the overall survival and time-to-progression disease was conducted in accordance with the method of Kaplan-Meier with log-rank test. For CTC-positive versus CTC-negative patients, the median time to progression disease was defined as the length of time from the T0 analysis (baseline) and the consequent beginning of chemotherapy, until disease progression. Probability values <0.05 were considered to be statistically significant.

## 3. Results

### 3.1. Assessment of the Induction of TGF-*β*1-Mediated EMT in A549 Cells

For the analysis of early EMT-like phenotypic change activation in the TGF-*β*1-mediated EMT processes in A549 cells, we assessed the morphology, immunophenotype, and gene expression characteristics of treated versus untreated cells.

**Figure 2:**
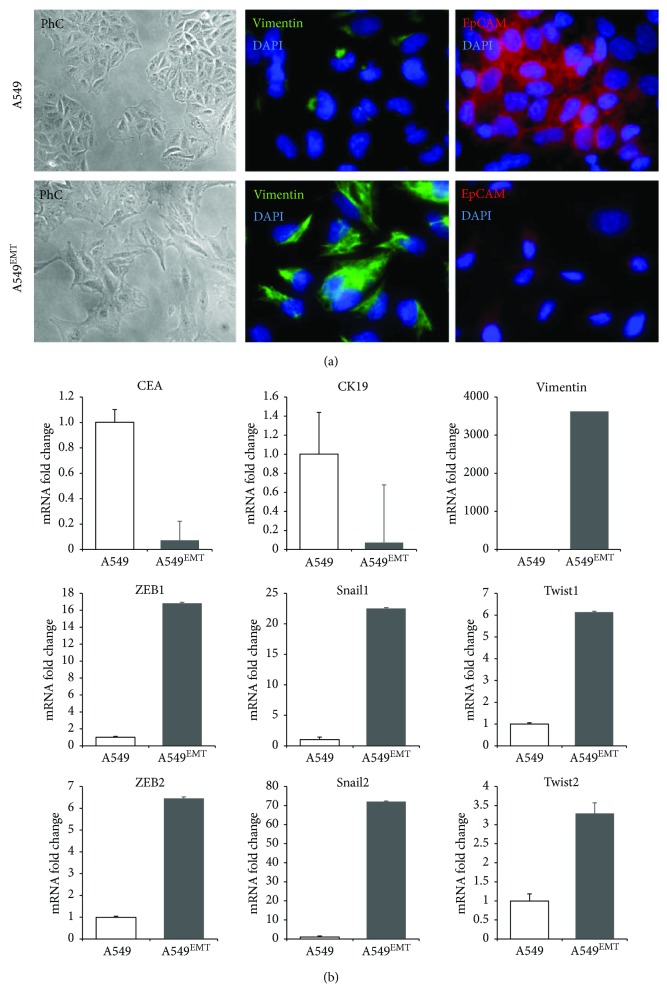
Representative micrographs showing the assessment of TGF-*β*1-mediated EMT in A549 cells. (a) The morphological analysis by differential interference contrast microscopy of the A549 cells exposed to TGF-*β*1 shows the acquisition of an elongated morphology with reduction of intercellular contacts compatible with EMT-like compared to unexposed A549 cells. (b) The immunofluorescence analysis reveals a dramatic reduction of EpCAM-positive cells (red fluorescence) in A549 cells exposed to TGF-*β*1 as compared to unexposed cells which retain the typical bordered staining. Furthermore, the A549 TGF-*β*1-treated cells showed a strong intracytoplasmic positivity for vimentin.

With a phase-contrast microscopy analysis, untreated A549 showed a typical epithelial morphology with clear cell-cell adhesion, while A549 TGF-*β*1-treated cells presented a reduction of intercellular contacts and a spindle-like morphology, suggestive of EMT. At the immunofluorescence assay, A549 TGF-*β*1-treated cells also showed a vimentin expression close to 100% and a severe reduction of epithelial adhesion molecule expression EpCAM that, in contrast, is widely expressed in untreated A549 (Figure 2(a)).

The induction of EMT TGF-*β*1-dependent on A549 cells was confirmed by RT-PCR analysis of mRNA expression levels for vimentin and for transcription factors Snail, Twist, and ZEB. The cycle threshold (Ct) comparison applied to calculate target gene expression changes induced by treatment showed a noticeable increase of the expression of all mesenchymal genes analyzed. In the A549-TGF-*β*1-treated cells, the mRNA fold increase amounted to over 3600 times for vimentin, about 17 and 6.5 times, respectively, for ZEB1 and ZEB2, 23 and 72 times for Snail1 and Snail2, and 6 and 3 times for Twist1 and Twist2 with respect to untreated A549 cells. The mRNA expression for CEA and CK19 was decreased with respect to the untreated A549 (Figure 2(b)).

### 3.2. Recovery Experiments of Spiked A549^EMT^ Cells and Reliability Evaluation of the RT-PCR Assay

Once confirmed, the induction of TGF-*β*1-mediated EMT in A549 cells, we spiked tenfold serial dilutions of A549^EMT^ in whole blood samples from healthy volunteers and we performed the recovery experiments as above to test the reliability and linearity of our detection method.

**Figure 3:**
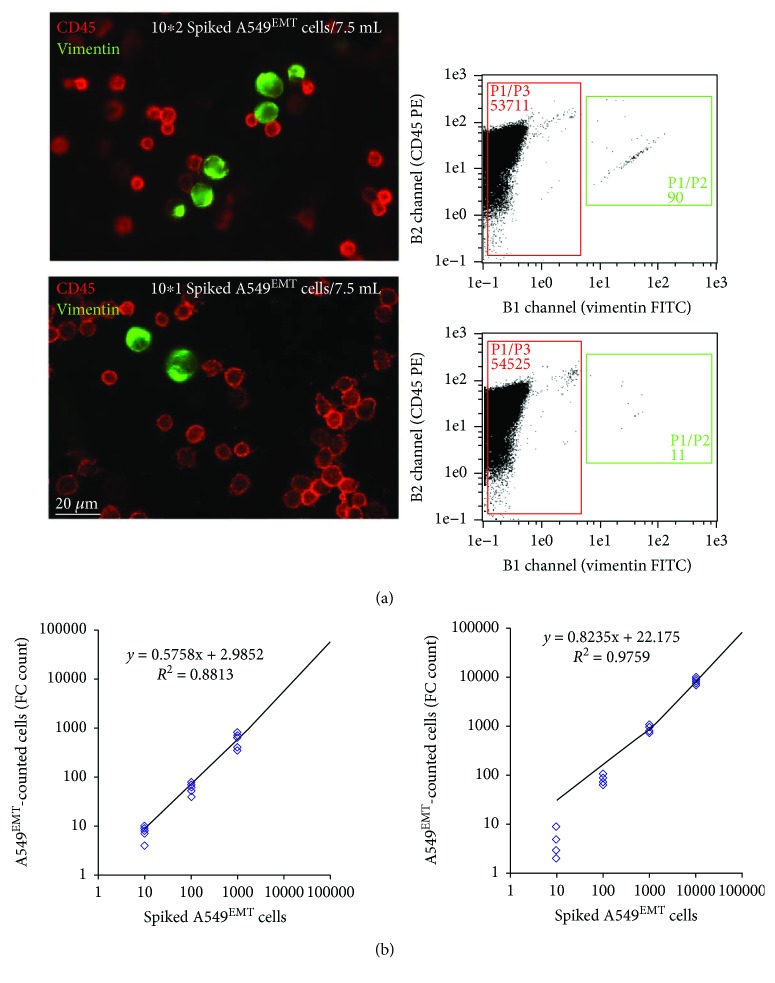
Recovery experiments of spiked A549^EMT^ cells and linearity assessment of control methods for cell count. (a) qIF and CF cell counts of spiked A549^EMT^ cells. All dilutions were analyzed in parallel; the mean recovery rates at lower dilutions (10^1^ and 10^2^) were of 61 ± 14% and 80 ± 17% for qIF and 84 ± 16% and 56 ± 32% for CF. (b) Evaluation of the linearity of qIF and CF cell counts. The linearity of both methods was verified by linear analysis of regression curve (*r*^2^: 0.88 and 0.97 for qIF and CF assays, resp.).

To count the spiked A549^EMT^ cells, all dilutions were analyzed in parallel by qIF and CF assays. The mean recovery rates at lower dilutions (10^1^ and 10^2^) were of 61 ± 14% and 80 ± 17% for qIF and 84 ± 16% and 56 ± 32% for CF (Figure 3(a)). The linearity of both methods was verified by linear analysis of regression curve (*r*^2^: 0.88 and 0.97 for qIF and CF assays, resp., Figure 3(b)).

**Figure 4:**
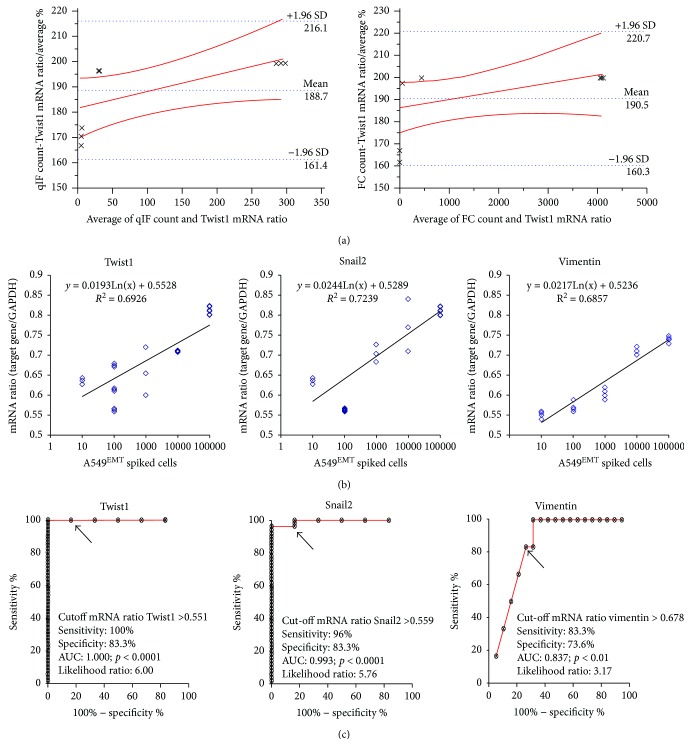
Reliability assessment of the RT-PCR assay. (a) Comparison between RT-PCR assay and control methods for cell counts. The reliability of RT-PCR method was assessed by Bland & Altman plot. At the different experimental points, the differences between methods were within mean ± 1.96 SD; therefore, the RT-PCR assay was interchangeable with both the qIF and CF assays. (b) Evaluation of the linearity of RT-PCR assay. A significant correlation between the number of spiked A549^EMT^ cells and levels of mRNA expression was found for all target genes, mainly for the expression of vimentin (*r*^2^: 0.68), Snail2 (*r*^2^: 0.72), and Twist1 (*r*^2^: 0.69). (*r* correlation coefficient for other target genes: Snail1 (*r*^2^: 0.68), Twist2 (*r*^2^: 0.68), ZEB1 (*r*^2^: 0.57), and ZEB2 (*r*^2^: 0.37)). (c) ROC curves and cutoff values for EMT-target genes (VIM: >0.678; sens 83.3, spec 73.6, likelihood 3.17, AUC 0.837, *P* < 0.01; Twist1: >0.551; sens 100, spec 83.3, likelihood 6.00, AUC 1.00, *P* < 0.0001; Twist2: >0.551, sens 100, spec 83.3, likelihood 6.00, AUC 1.00, *P* < 0.0001; Snail1: >0.718, sens 74.0, spec 83.3, likelihood 4.44, AUC 0.77, *P* < 0.05; Snail2: >0.559, sens 96.0, spec 83.3, likelihood 5.76, AUC 0.993, *P* < 0.0001; ZEB1: >0.765, sens 72.0, spec 83.3, likelihood 4.32, AUC 0.736, *P* < 0.05; ZEB2: > 0.88, sens 92.0, spec 83.3, likelihood 5.52, AUC 0.923, *P* < 0.001).

The reliability of RT-PCR method was assessed by Bland & Altman plot. The expression levels of all target genes were compared to qIF and CF cell counts. The RT-PCR assay was found consistent and interchangeable with both CTC count methods (Figure 4(a)). Finally, the linearity of RT-PCR assay was confirmed by linear regression analysis; a significant correlation between the number of spiked A549^EMT^ and levels of mRNA expression was found for all target genes, mainly for the expression of vimentin, Snail2, and Twist1 (Figure 4(b)).

### 3.3. Detection of CTCs in NSCLC Patients

**Figure 5:**
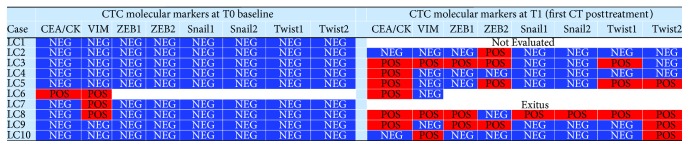
CTC-positive samples (red) with mRNA levels higher than the cutoff of epithelial and/or at least an EMT-related gene.

We evaluated peripheral blood samples from ten patients with metastatic NSCLC and ten healthy volunteers. Clinical and histopathological characteristics of patients are summarized in Table 2. Performance status (PS) was classified according to the Eastern Cooperative Oncology Group (ECOG) score. Putative tumor cells recovered after immunomagnetic depletion of CD45^+^ cells were analyzed by RT-PCR. Samples with both CEA and CK19 and/or one of the EMT-related genes (vimentin and/or EMT transcription factors) expressed above the cutoff levels (Figure 4(c)) were considered positive for CTCs. At baseline (Figure 5), three of ten samples were positive for CTCs; particularly, a patient (LC6) was found positive for CTCs with mixed epithelial and mesenchymal molecular profile, while two patients (LC7 and LC8) were positive for CTCs with mesenchymal molecular profile. All the subjects from the control group showed mRNA levels below the cutoff.

After four cycles of first-line platinum-based chemotherapy (T1, median time 140 days from baseline), two patients were excluded from the study (LC1 received treatment at another centre, and LC7 died at the early steps of the current study). By this time, the percentage of patients with CTC positivity showed a strong increase (T1; Figure 5): two patients showed positivity for CTCs with an epithelial profile (LC4 who was negative at T0 and LC6), two showed positivity for CTCs with mixed profile (LC3 and LC9), and three showed positivity for CTCs with mesenchymal profile (LC5, LC8, and LC10).

### 3.4. Prognostic Significance of Epithelial and/or Mesenchymal Phenotype Expression in CTCs

Three patients with CTC positivity at baseline showed a progression faster than the counterpart with a negative CTC count (median time to progression: 190 versus 275 days; *P* = NS). The positivity for CTCs expressing mesenchymal phenotype after the first four cycles of chemotherapy treatment seems to be related to a more unfavorable prognostic trend: patients who develop CTCs^EMT^-positive at the T1 showed a progression or a faster progression time (median time to progression: 1 day for CTCs^EMT^-positive versus 90 days for CTCs^EMT^-negative patients in T1; *P* = NS) and showed a notable shorter overall survival (median time of OS: 148 days for CTCs^EMT^-positive versus 350 days for CTCs^EMT^-negative patients in T1; *P* = NS).

### 3.5. Dynamic Variations in the CTC Molecular Profile: Clinical Outcomes and Therapeutic Response

Three patients still alive were treated with taxane-based chemotherapy and underwent to a third levy during the second posttreatment CT scan (T2) to verify a possible association between the positivity for CTCs^EMT^ and the therapeutic response or progression.

**Figure 6:**
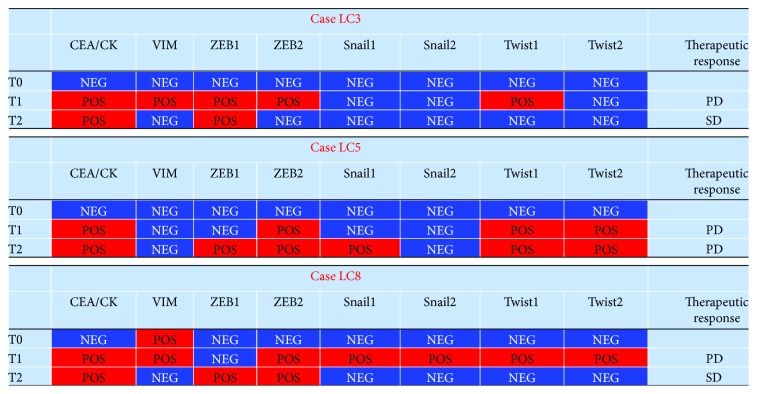
Dynamic variations of CTC molecular profile in still living patients at T1 and T2 evaluation with respect to clinical outcomes and therapeutic response (SD: stable disease, PD: progression disease).

The LC3 patient had a stable disease at T2 while previously positive for CTCs, and he showed a partial reversion of the molecular profile (a negative expression of vimentin, Twist1, and ZEB2, which were previously positive at T1). A partial reversion of CTC mesenchymal phenotype was also found in the LC8 patient who was also clinically stable at T2. Instead, the LC5 patient, who had a clinical progression of disease at T2, besides confirming the positivity for ZEB2 and Twist1 and 2 presented an accentuated tendency toward the expression of EMT markers with a positive status of ZEB1 and Snail1 (Figure 6).

## 4. Discussion

EMT leads to epithelial cell dedifferentiation and acquisition of cell motility and invasiveness through the rearrangement of cell junctions and loss of cell adhesion factors; this mechanism, which is involved in organogenesis and wound healing, is implicated in the dissemination of neoplastic cells, and it has also been associated with the aggressiveness of the tumor and the migration of malignant cells from the primary mass [2–4]. Despite a recent meta-analysis on the significance of CTCs in lung cancer patients that has established the prognostic value of CTC positivity based on detection of epithelial markers [32], these standard methods can easily fail when the partial loss of epithelial markers due to epithelial-mesenchymal plasticity leads to an underestimation of the CTCs^EMT^ with hybrid or mesenchymal phenotype [16, 18–20, 33].

The usefulness of assessing other tumor markers and/or the coexpression of different target genes has been clearly documented [14, 25–27, 34, 35]. Therefore, in a setting of metastatic NSCLC patients, we tried to optimize a method of enrichment for CTC detection based on negative immunomagnetic selection of CTCs associated with a RT-PCR analysis for the expression of epithelial (CEA and CK19) and EMT-related markers such as vimentin and transcription factors (Snail1 and 2, ZEB1 and 2, and Twist1 and 2).

The choice of EMT-related genes comes from the fact that ZEB and Snail act directly on E-cadherin gene promoter sequences inhibiting its expression, while Twist genes act indirectly because they have a crucial role on maintaining stem properties of cancer cells through activation of beta catenin and AKT pathway [36, 37]. Snail genes seem to be directly involved in EMT in many types of cancer by inducing cancer cells to enter in the systemic circulation [38]; ZEB and Twist are involved in EMT, and they induce stem cell neoplastic properties [39, 40]. In the NSCLC patients, the overexpression of Snail is associated with reduced survival [41].

The aim of the study is to show that an alternative CTC detection approach focused in the recognition and association of epithelial and mesenchymal markers directly involved in EMT can lead to a better recovery of the CTCs. The feasibility of this method was confirmed by our experiments on A549^EMT^ cell lines by linking spiked A549^EMT^, recovered cells, and mRNA expression. After that, we evaluated the same approach on blood samples from our series of NSCLC patients.

At the time of the enrolment, 30% of the patients were found to be positive for CTCs, and in two-thirds of cases, this positivity was related exclusively to the expression of mesenchymal target genes. The positivity for CTCs seems to be increased significantly in T1 achieving a percentage close to 90%, and in more than half of the patients (62.5%), there was a positivity in the expression of mesenchymal markers. Finally, two patients express exclusively mRNA of a transcription factor related to EMT, without any expression of vimentin, CEA, and CK19. This strong increase of mesenchymal target genes in T1 is probably related to chemotherapy; it is possible that chemotherapeutic drugs directly promote EMT [42] or a drug-induced debulking where highly dividing nonmesenchymal cells die sparing the more quiescent mesenchymal-like cells [43]. Anyhow, the development of CTC positivity after therapy is an unfavorable prognostic factor; in fact, we observed that the positivity for CTCs^EMT^ during chemotherapy is associated with faster progression and shorter overall survival.

Our data lack of any statistical ambition, but these results are only anecdotal and have shown how chemotherapy response and a stable disease are associated with phenotypic transition from mesenchymal to epithelial markers (MET), while no response and disease progression are associated with expression of markers related to EMT. Nel et al. found similar results in their study where CTCs were negatively enriched by hematopoietic cell depletions from blood samples, and then they were further characterized by multi-immunofluorescence staining against pan-CK, EpCAM, N-cadherin, stem cell marker CD133, CD45, and nuclear counterstain DAPI: the presence of mesenchymal N-cadherin-positive cells was associated to shortened PFS (5 versus 8 months, *P* = 0.03, HR = 2.63) [44]. In that study, however, they took 20 mL of blood from 43 patients with NSCLC at various stages of disease (56% stage IV) using, after the negative selection, immunofluorescence, a method with a poor sensitivity. In another study in NSCLC patients, CTCs were highlighted by CK7 mRNA expression measured by RT-PCR; there was not a significant association between CK7 mRNA levels and the response to chemotherapy or the survival [45]. Based on our data, we think this contrasting result is due to the use of only one epithelial marker and so it emphasizes how the expression of mesenchymal markers in combination with epithelial ones can significantly improve the CTCs detection. These conclusions are consistent with the current literature, where the emergence of EMT may explain the onset of resistance to chemotherapeutic approaches and thus represent a particularly valuable target to be considered in clinical practice to predict worse prognoses [46–48]. In a meta-analysis assessing the predictive and prognostic significance of CTCs in lung cancer patients treated with chemotherapy, the authors found that patients converted from CTC-negative to CTC-positive and patients persistently CTC-positive had a worse disease control rate compared to those converted from CTC-positive to CTC-negative or to patients persistently negative [49].

## 5. Conclusions

At the current time, no procedures are considered “the gold standard” for CTC evaluation as a prognostic or predictive marker. It is because many studies recruited too few patients, the methods used are varied, and the findings are contradictory or inconclusive [50]. Limits of the extensive use of CTC assay in clinical practice are various. There is not a consensus about requirements necessary and sufficient to define an event as CTCs in the blood. The available technologies for CTC detection are often very expensive and require specific equipment and qualified technical personnel. In addition, the EpCAM-dependent strategies lack of sensitivity, so the more aggressive and undifferentiated CTC EMT will be underestimated and cannot be correctly quantified.

Our study covers a hot topic in the field of translational biology; in fact, it proposes an inexpensive, simple, and reliable method to highlight the CTCs with EMT phenotype. The RT-PCR method does not require highly specialized laboratories, and the costs are not the “stumbling block” [51]. Moreover, although the results are based on a small number of patients, it highlighted that EMT may occur in CTCs, CTC count should not be just based on EpCAM detection, and EMT-related gene expression by CTCs could have a significance in terms of prognosis and response to chemotherapy.

## Figures and Tables

**Table 1:** Primers used for target and housekeeping genes.

GAPDH	For 5′-CATCAGCAATGCCTCCTGCAC-3′
GAPDH	Rev 5′-GTCATGAGTCCTTCCACGATACCAA-3′
CEA	For 5′-AGGACAGAGCAGACAGCAGAG-3′
CEA	Rev 5′-GGTTCCAGAAGGTTAGAAGTGAGG-3′
CK19	For 5′-CCTGACACCATTCCTCCCTTC-3′
CK19	Rev 5′-CCGACGACTGGCGATAGC-3′
ZEB1	For 5′-GGGAGGAGCAGTGAAAGAGA-3′
ZEB1	Rev 5′-TTTCTTGCCCTTCCTTTCTG-3′
ZEB2	For 5′-AAGCCAGGGACAGATCAGC-3′
ZEB2	Rev 5′-CCACACT CTGTGCATTTGAACT-3′
Snail1	For 5′-GCTGCAGGACTCTAATCCAGA-3′
Snail1	Rev 5′-ATCTCCGGAGGTGGGATG-3′
Snail2	For 5′-TGGTTGCTTCAAGGACACAT-3′
Snail2	Rev 5′-GCAAATGCTCTGTTGCAGTG-3′
Twist1	For 5′-AGCTACGCCTTCTGGTCT-3′
Twist1	Rev 5′-CCTTCTCTGGAAACAATGACATC-3′
Twist2	For 5′-CATGTCCGCCTCCCACTA-3′
Twist2	Rev 5′-GCATCATTCAGAATCTCCTCCT-3′
Vimentin	For 5′-AATCCAAGTTTGCTGACCTCTCTG-3′
Vimentin	Rev 5′-TCATTGGTTCCTTTAAGGGCATCC-3′

**Table 2:** Clinical and histopathological characteristics of ten non-small cell lung cancer patients.

Factors	Subgroup	*N*	%
Median age at baseline		69.9 y (45–70)	

Sex	Male	6	60
	Female	4	40

Smoker	Yes	5	50
	No	2	20
	Unknown	3	30

ECOG PS	0-1	10	100
	2	0	0

Histopathology	Adenocarcinoma	9	90
	Squamous cell	1	10

Mutational status	EGFR mutation	0	0
	ALK translocation	1	10
	ROS1 translocation	1	10
	None	8	80

Metastasis location	Bone	1	10
	Liver	1	10
	Contralateral lung	4	40
	Adrenal gland	1	10
	Brain	3	30

Chemotherapy	CDDP-pemetrexed	7	70
	CDDP-gemcitabine	2	20
	CDDP-taxotere	1	10

## References

[1] Allard W. J., Matera J., Miller M. C. (2004). Tumor cells circulate in the peripheral blood of all major carcinomas but not in healthy subjects or patients with nonmalignant diseases. *Clinical Cancer Research*.

[2] Lamouille S., Xu J., Derynck R. (2014). Molecular mechanisms of epithelial-mesenchymal transition. *Nature Reviews Molecular Cell Biology*.

[3] Sleeman J. P., Thiery J. P. (2011). SnapShot: the epithelial-mesenchymal transition. *Cell*.

[4] Bàrdos J. I., Ashcroft M. (2005). Negative and positive regulation of HIF-1: a complex network. *Biochimica et Biophysica Acta (BBA) - Reviews on Cancer*.

[5] Moustakas A., Heldin C. H. (2007). Signaling networks guiding epithelial-mesenchymal transitions during embryogenesis and cancer progression. *Cancer Science*.

[6] Sánchez-Tilló E., Liu Y., de Barrios O. (2012). EMT-activating transcription factors in cancer: beyond EMT and tumor invasiveness. *Cellular and Molecular Life Sciences*.

[7] Hanahan D., Weinberg R. A. (2011). Hallmarks of cancer: the next generation. *Cell*.

[8] Yu M., Stott S., Toner M., Maheswaran S., Haber D. A. (2011). Circulating tumor cells: approaches to isolation and characterization. *The Journal of Cell Biology*.

[9] Caixeiro N. J., Kienzle N., Lim S. H. (2014). Circulating tumour cells—a bona fide cause of metastatic cancer. *Cancer Metastasis Reviews*.

[10] Budd G. T., Cristofanilli M., Ellis M. J. (2006). Circulating tumor cells versus imaging--predicting overall survival in metastatic breast cancer. *Clinical Cancer Research*.

[11] Cohen S. J., Punt C. J. A., Iannotti N. (2008). Relationship of circulating tumor cells to tumor response, progression-free survival, and overall survival in patients with metastatic colorectal cancer. *Journal of Clinical Oncology*.

[12] de Bono J. S., Scher H. I., Bruce Montgomery R. (2008). Circulating tumor cells predict survival benefit from treatment in metastatic castration-resistant prostate cancer. *Clinical Cancer Research*.

[13] Torre L. A., Bray F., Siegel R. L., Ferlay J., Lortet-Tieulent J., Jemal A. (2015). Global cancer statistics, 2012. *CA: a Cancer Journal for Clinicians*.

[14] Krebs M. G., Sloane R., Priest L. (2011). Evaluation and prognostic significance of circulating tumor cells in patients with non-small-cell lung cancer. *Journal of Clinical Oncology*.

[15] Hanssen A., Loges S., Pantel K., Wikman H. (2015). Detection of circulating tumor cells in non-small cell lung cancer. *Frontiers in Oncology*.

[16] Tang Y., Qiao G., Xu E., Xuan Y., Liao M., Yin G. (2017). Biomarkers for early diagnosis, prognosis, prediction, and recurrence monitoring of non-small cell lung cancer. *OncoTargets and Therapy*.

[17] Alix-Panabières C., Pantel K. (2013). Circulating tumor cells: liquid biopsy of cancer. *Clinical Chemistry*.

[18] Grover P. K., Cummins A. G., Price T. J., Roberts-Thomson I. C., Hardingham J. E. (2014). Circulating tumour cells: the evolving concept and the inadequacy of their enrichment by EpCAM-based methodology for basic and clinical cancer research. *Annals of Oncology*.

[19] Van der Toom E. E., Verdone J. E., Gorin M. A., Pienta K. J. (2016). Technical challenges in the isolation and analysis of circulating tumor cells. *Oncotarget*.

[20] Bozzetti C., Quaini F., Squadrilli A. (2015). Isolation and characterization of circulating tumor cells in squamous cell carcinoma of the lung using a non-EpCAM-based capture method. *PLoS One*.

[21] Lecharpentier A., Vielh P., Perez-Moreno P., Planchard D., Soria J. C., Farace F. (2011). Detection of circulating tumour cells with a hybrid (epithelial/mesenchymal) phenotype in patients with metastatic non-small cell lung cancer. *British Journal of Cancer*.

[22] Yin J., Wang Y., Yin H. (2015). Circulating tumor cells enriched by the depletion of leukocytes with bi-antibodies in non-small cell lung cancer: potential clinical application. *PLoS One*.

[23] Yu N., Zhou J., Cui F., Tang X. (2015). Circulating tumor cells in lung cancer: detection methods and clinical applications. *Lung*.

[24] Pailler E., Adam J., Barthélémy A. (2013). Detection of circulating tumor cells harboring a unique *ALK* rearrangement in *ALK*-positive non-small-cell lung cancer. *Journal of Clinical Oncology*.

[25] Ilie M., Long E., Butori C. (2012). *ALK*-gene rearrangement: a comparative analysis on circulating tumour cells and tumour tissue from patients with lung adenocarcinoma. *Annals of Oncology*.

[26] Pailler E., Auger N., Lindsay C. R. (2015). High level of chromosomal instability in circulating tumor cells of *ROS*_1_-rearranged non-small-cell lung cancer. *Annals of Oncology*.

[27] Maheswaran S., Sequist L. V., Nagrath S. (2008). Detection of mutations in *EGFR* in circulating lung-cancer cells. *New England Journal of Medicine*.

[28] Leone L., Mazzetta F., Martinelli D. (2016). *Klebsiella pneumoniae* is able to trigger epithelial-mesenchymal transition process in cultured airway epithelial cells. *PLoS One*.

[29] Kasai H., Allen J. T., Mason R. M., Kamimura T., Zhang Z. (2005). TGF-*β*1 induces human alveolar epithelial to mesenchymal cell transition (EMT). *Respiratory Research*.

[30] Romiti A., Raffa S., Di Rocco R. (2014). Circulating tumor cells count predicts survival in colorectal cancer patients. *Journal of Gastrointestinal and Liver Diseases*.

[31] Rossi Del Monte S., Ranieri D., Mazzetta F. (2012). Free peritoneal tumor cells detection in gastric and colorectal cancer patients. *Journal of Surgical Oncology*.

[32] Xu T., Shen G., Cheng M., Xu W., Shen G., Hu S. (2017). Clinicopathological and prognostic significance of circulating tumor cells in patients with lung cancer: a meta-analysis. *Oncotarget*.

[33] Wu S., Liu S., Liu Z. (2015). Classification of circulating tumor cells by epithelial-mesenchymal transition markers. *PLoS One*.

[34] Hanssen A., Wagner J., Gorges T. M. (2016). Characterization of different CTC subpopulations in non-small cell lung cancer. *Scientific Reports*.

[35] Micalizzi D. S., Haber D. A., Maheswaran S. (2017). Cancer metastasis through the prism of epithelial-to-mesenchymal transition in circulating tumor cells. *Molecular Oncology*.

[36] Lì J. L., Zhou B. H. P. (2011). Activation of *β*-catenin and Akt pathways by twist are critical for the maintenance of EMT associated cancer stem cell-like characters. *BMC Cancer*.

[37] Roth B., Jayaratna I., Sundi D. (2017). Employing an orthotopic model to study the role of epithelial-mesenchymal transition in bladder cancer metastasis. *Oncotarget*.

[38] Wang Y., Shi J., Chai K., Ying X., Zhou B. P. (2013). The role of snail in EMT and tumorigenesis. *Current Cancer Drug Targets*.

[39] Galván J. A., Helbling M., Koelzer V. H. (2015). TWIST1 and TWIST2 promoter methylation and protein expression in tumor stroma influence the epithelial-mesenchymal transition-like tumor budding phenotype in colorectal cancer. *Oncotarget*.

[40] Peinado H., Olmeda D., Cano A. (2007). Snail, Zeb and bHLH factors in tumour progression: an alliance against the epithelial phenotype?. *Nature Reviews Cancer*.

[41] Yanagawa J., Walser T. C., Zhu L. X. (2009). Snail promotes CXCR2 ligand-dependent tumor progression in non-small cell lung carcinoma. *Clinical Cancer Research*.

[42] Li Q. Q., Chen Z. Q., Cao X. X. (2011). Involvement of NF-*κ*B/miR-448 regulatory feedback loop in chemotherapy-induced epithelial-mesenchymal transition of breast cancer cells. *Cell Death and Differentiation*.

[43] Xiao D., He J. (2010). Epithelial mesenchymal transition and lung cancer. *Journal of Thoracic Diseases*.

[44] Nel I., Jehn U., Gauler T., Hoffmann A. C. (2014). Individual profiling of circulating tumor cell composition in patients with non-small cell lung cancer receiving platinum based treatment. *Translational Lung Cancer Research*.

[45] Koren A., Sodja E., Rijavec M. (2015). Prognostic value of cytokeratin-7 mRNA expression in peripheral whole blood of advanced lung adenocarcinoma patients. *Cellular Oncology*.

[46] Singh A., Settleman J. (2010). EMT, cancer stem cells and drug resistance: an emerging axis of evil in the war on cancer. *Oncogene*.

[47] Polyak K., Weinberg R. A. (2009). Transitions between epithelial and mesenchymal states: acquisition of malignant and stem cell traits. *Nature Reviews Cancer*.

[48] Thiery J. P., Acloque H., Huang R. Y., Nieto M. A. (2009). Epithelial-mesenchymal transitions in development and disease. *Cell*.

[49] Wu Z. X., Liu Z., Jiang H. L., Pan H. M., Han W. D. (2016). Circulating tumor cells predict survival benefit from chemotherapy in patients with lung cancer. *Oncotarget*.

[50] Zhang Z., Ramnath N., Nagrath S. (2015). Current status of CTCs as liquid biopsy in lung cancer and future directions. *Frontiers in Oncology*.

[51] Lianidou E. S., Markou A., Strati A., Cote R., Datar R. (2016). Molecular assays for the detection and molecular characterization of CTCs. *Circulating Tumor Cells. Current Cancer Research*.

